# High-fructose diet induces depressive-like behaviors and short-term memory deficits through hippocampal neurogenesis impairment via neural stem cell dysfunction

**DOI:** 10.1186/s12986-025-01043-7

**Published:** 2025-12-02

**Authors:** Qiaona Wang, Yongfa Wang, Yuefeng Hu, Pengfei Xie, Fan Li, Ruoyu Mu, Zhenjie Feng, Feng Zhou, Chuanfeng Tang

**Affiliations:** 1 State Key Laboratory of Technologies for Chinese Medicine Pharmaceutical Process Control and Intelligent Manufacture, Nanjing University of Chinese Medicine, Nanjing, 210023 People’s Republic of China; 2 School of Food Science, Nanjing Xiaozhuang University, Nanjing, 211171 People’s Republic of China; 3 School of Food Science and Pharmaceutical Engineering, Nanjing Normal University, Nanjing, 210023 People’s Republic of China

**Keywords:** High-fructose diet, Neural stem cells, Energy metabolism, Apoptosis, Ferroptosis

## Abstract

**Background:**

Neural stem cells (NSCs), crucial for brain function and repair, are disrupted by high-fructose diet (HFrD) in proliferation and survival, linking to neurogenesis impairment and neuropsychiatric risks. Mechanistic insights remain undefined.

**Methods:**

Comprehensive behavioral assessments were conducted on HFrD mice, including the tail suspension test (TST) and sucrose preference test (SPT) for depressive-like behaviors, elevated plus maze (EPM) and open field test (OFT) for anxiety-like behaviors, as well as novel object recognition (NOR) and Morris water maze (MWM) for cognition. Hippocampal NSCs and newborn neurons were quantified by immunofluorescence, and fructose-treated NE-4C cells underwent RNA sequencing (RNA-seq) analysis coupled with measurements of proliferation, apoptosis and ferroptosis markers.

**Results:**

HFrD mice showed depressive-like behaviors without anxiety-like behaviors, and exhibited impaired short-term memory in NOR but did not show impaired spatial memory in MWM. Decreased number of hippocampal NSCs and newborn neurons were observed, suggesting impaired neurogenesis. In vitro, fructose-treated NE-4c exhibited altered gene expression profiles, with PCA showing distinct clustering between treated and control groups. Further analysis (GO, KEGG, GSEA) indicated enrichment in energy metabolism pathways, including mitochondrial ATP synthesis (e.g., downregulated ATP5E, ATP5H). Consistently, intracellular ATP levels were elevated, indicating metabolic dysregulation. Further experiments demonstrated that high fructose promoted NSC proliferation via p53/Wnt pathways (upregulated CyclinA2, CDK1) while concurrently inducing apoptosis (BAX, P53 upregulation) and ferroptosis (reduced GPX4, elevated ROS, and lipid peroxidation).

**Conclusion:**

This study elucidates the mechanistic link between HFrD-induced metabolic disruption and NSC dysfunction, providing novel insights into the pathogenesis of fructose-associated neuropsychiatric disorders.

**Supplementary Information:**

The online version contains supplementary material available at 10.1186/s12986-025-01043-7.

## Introduction

The modern diet, characterized by excessive intake of high-fructose corn syrup and other fructose-rich foods, poses significant risks to metabolic and neurological health [1, 2]. Sustained consumption of a high-fructose diet (HFrD) has been implicated in the development of multiple neuropsychiatric conditions, such as mood and memory deficits, via the dysregulation of neurogenesis and neuroinflammatory pathways [2–5]. The hippocampus, which is a brain region pivotal for mood regulation and memory consolidation, depends fundamentally on neural stem cells (NSCs). The capacity of NSCs for self-renewal and differentiation into new neurons sustains hippocampal neurogenesis [6, 7].

Recent studies have highlighted the vulnerability of NSCs to metabolic stress, including aberrant energy metabolism, oxidative damage, and dysregulated cell death pathways [8–10]. The relationship between HFrD and NSC biology remains poorly understood. The studies by Liu et al. [11]. and Li et al. [12]. showed that 12-week HFrD impairs hippocampal neurogenesis in rats and mice. Under physiological conditions, active neurogenesis occurs mainly in two specific brain regions, one of which is the subgranular zone (SGZ) within the hippocampal dentate gyrus. The SGZ is the most active site for neurogenesis in adult mammals, where newly generated neurons can stably integrate into local neural circuits and directly support core functions, such as learning, memory, and emotional regulation [13, 14]. In contrast, adult neurogenesis in the prefrontal cortex is extremely limited, and neurogenesis in the striatum is only slightly activated after injury [15]. Our previous findings demonstrated that 8-week HFrD exposure depletes hippocampal NSC numbers and impairs neurogenesis in mice [3]. However, the mechanisms underlying HFrD-induced NSC dysfunction, which may involve metabolic disruption, apoptosis, or other pathways, remain elusive. This fundamental gap impedes a mechanistic understanding of how dietary factors promote neuropsychiatric disorders via NSC impairment.

Energy metabolism is a fundamental biological process essential for maintaining physiological functions and is critically involved in both health and disease states [16]. Energy metabolism encompasses both canonical ATP-generating pathways, such as oxidative phosphorylation and glycolysis, and coordinated processes including mitochondrial oxidative stress regulation and lipid metabolic homeostasis [17, 18]. Disorders of energy metabolism regulate the processes of cell proliferation and death [19, 20]. The self-renewal capacity of NSCs is critical for maintaining the hippocampal NSC pool and neurogenesis [21]. However, aberrant NSC proliferation also can paradoxically deplete the NSC pool [22]. Xu et al. demonstrated that Mysm1 knockout promotes both NSC excessive proliferation and apoptosis, consequently resulting in neural stem cell pool exhaustion [23]. Similarly, arginase-II deficiency mice exhibited NSC overactivation, accelerating pool exhaustion and impairing neurogenesis [22]. However, the impact of HFrD on NSC energy metabolism and proliferative dynamics remains poorly characterized.

Cell death including apoptosis and ferroptosis, are critical regulators of NSC population dynamics and consequently impact neurogenesis [24]. Apoptosis existing studies have demonstrated that high fructose exposure induces hepatocyte apoptosis accompanied by oxidative stress and inflammation [25]. However, the effects of HFrD on NSC-specific apoptotic activation remain largely unexplored. Apoptosis is governed by conserved molecular players including p53, BAX, BCL-XL, and caspase-3, which collectively mediate stress signal integration and mitochondrial-dependent caspase activation. This cascade progresses from p53-initiated signaling through BAX/BAK oligomerization-induced MOMP to effector caspase proteolysis [24, 26].

Ferroptosis has emerged as another critical cell death pathway, defined by its iron-dependent nature and driven primarily by glutathione peroxidase 4 (GPX4) inactivation and subsequent lipid peroxidation [11]. This unique form of cell death further manifests through mitochondrial dysfunction, reactive oxygen species (ROS) accumulation, and dysregulation of iron homeostasis, including elevated labile iron pools [16]. Key ferroptosis regulators also include iron metabolism proteins FTH1 and antioxidant defenses NRF2, SOD1 et al. Notably, cellular energy metabolism activities such as glycolysis, pentose phosphate pathway, and tricarboxylic acid cycle are involved in the regulation of key ferroptosis markers [27].

Therefore, cell death is regulated by metabolism, but systematic investigations are lacking in HFrD-induced NSC death. In this study, we systematically investigate fructose-induced disturbances in both energy metabolism and multiple programmed cell death pathways (apoptosis and ferroptosis) in hippocampal NSCs. We provide novel evidence that HFrD not impairs mitochondrial energy generation but also triggers concurrent apoptotic and ferroptotic cascades, leading to NSC pool exhaustion. These findings address a significant gap in understanding how dietary fructose contributes to hippocampal dysfunction via NSC-specific metabolic and cytotoxic mechanisms.

## Methods

### Animals: high-fructose diet procedure

C57BL/6J male mice at age of 6 week were purchased from GemPharmatech Co., Ltd. (Jiangsu, China) and kept in a specific pathogen-free facility in Animal Research Center of Nanjing University of Chinese Medicine. The sample size was determined using G*Power analysis [28] with the following parameters: α error probability = 0.05, statistical power (1 - β) = 0.80, effect size = 0.25, number of groups = 3, number of measurements = 4, correlation among repeated measures = 0.5, and non-sphericity correction ε = 1. The analysis yielded a minimum sample size of 36 rats. To accommodate potential attrition due to health or technical issues and to ensure equal group allocation, a 20% buffer was applied, resulting in a final total of 45 rats included in the study. Mice were acclimated to the environment in Animal Research Center for 2 weeks.Then mice were fed with a standard diet (normal control group, CTL, 15 mice, XTCON50B, Xietong Shengwu, Jiangsu, China), or a 60% high-fructose diet for 6 weeks (Fru6W, 15 mice, XT704, Xietong Shengwu) or 12 weeks (Fru12W, 15 mice). The animal husbandry and experimental procedures were approved by the Animal Ethical and Welfare Committee of Nanjing University of Chinese Medicine and Nanjing University (Approval No. 202406A042 and IACUC-2404014, China).

## Open field test (OFT)

OFT was performed in an open field box, which has dimensions of 50 cm in length, width, and height, wherein all mice were placed close to against one side of the box wall. Mice were allowed a 5-minute habituation period in the open field arena prior to formal testing to reduce novelty-induced anxiety. The mouse activity area was divided into 9 equal-sized squares, with three distinct regions marked: the corners, the edges, and the center. During 10 min, Supermaze software was used to record time spent and distance travelled in the open field box, including arena perimeter, center, and total distance travelled. After each trial, the open field boxes were cleaned with a 75% ethanol to eliminate odor cues.

## Elevated plus maze (EPM)

The EPM is made of white acrylic panels and consists of two open arms and two enclosed arms arranged in a cross-shaped layout. The length of each arm is 50 cm, the width is 10 cm, and the height of the enclosed arms is 15 cm to ensure sufficient concealment. Mice were transferred to the test room (maintained at the same temperature as the housing facility) 30 min prior to testing [28]. Mice were individually positioned at the center of a plus-shaped maze elevated 50 cm above the floor. Each mouse was gently placed in the center of the maze, with its back facing the experimenter, and its head oriented toward the open arms. Then, quickly and quietly leave the area. Activate the VisuTrack analysis software to record the percentage of time and number of entries the mouse spends in the open arms during the next 10 min. Between trials, the maze was thoroughly cleaned with 75% ethanol to eliminate olfactory cues.

## Sucrose preference test (SPT)

After 6 or 12 consecutive weeks of high fructose diet and medication treatment, sweet taste preference was detected by SPT in mice. Mice were individually housed for 3 days for adaptation before the test. Two bottles, one containing 1% sucrose solution and the other plain water, were placed in each cage. Their positions were switched every 24 h over a 48-hour period to avoid side preference. As described in previous study [7], each mouse was isolated and then accented to drinking either normal water or 1% sucrose solution. Twenty-four hours (h) of water deprivation before sweet taste preference test, each mouse was re-provided with two bottles for ensuing 2 h.

## Forced swim test (FST)

FST was conducted to assess depressive-like behavior in mice. During the swimming adaptation phase, the mice were placed in a swimming environment for 5 min one day before the formal FST. Formal experiment on the second day, mice were individually placed into glass cylinders (50 cm high × 20 cm internal diameter) filled with water (23–25 °C) to a depth of 15 cm. Test sessions were recorded by a video camera positioned directly front the cylinders. A well-trained observer analyzed these videotapes for the duration of mouse floating, swimming, and struggling with Supermaze software during the 6 min test period [29].

### Tail suspension test (TST)

TST was conducted to assess depressive-like behavior in mice. Briefly, each mouse was acoustically and visually isolated and suspended by the tail with adhesive tape (approximately 50 cm above the floor) in a sound-isolated chamber. The 6-minute session was recorded, and the immobility time was quantified by trained investigators blinded to the experimental groups using Supermaze software [30].

## Novel object recognition test (NOR)

The NOR was conducted to evaluate short-term memory function in mice according to standardized cognitive testing procedures. Mice placed in one of the large apparatus compartments (length, width, and height: 50 × 50 × 50 cm). Mice explored and habituated to this compartment for 5 min. The same objects were fixed in the NOR device in the first phase of NOR (one object was named “Old’’ because it would continue to be used in the second phase of NOR, and another was named “Same”). After 1 h, in the second phase of NOR, the object named “same” was replaced with a novel object with a different color and shape (the novel object was named “Novel”). After each trial, the objects and boxes were cleaned with a 70% ethanol to eliminate odor cues. The whole process of the test was recorded by the camera located directly above. The exploration time and number of each object were analyzed with Supermaze software [31].

## Morris water maze (MWM) assessment of Spatial learning and memory

Spatial learning and memory were evaluated in the MWM, a circular pool (120 cm diameter, 45 cm height) filled with temperature-controlled opaque water (22 ± 1 °C). The training paradigm included an initial visible platform session on Day 1 to establish baseline performance, followed by four days of hidden platform training (Days 2–5) with four daily trials (60 s maximum duration, 30 min inter-trial intervals) starting from pseudorandom positions. Spatial memory was assessed on Day 6 via a 60 s probe trial, with the platform removed, during which we quantified search strategy efficiency by measuring time spent in the target quadrant, number of platform location crossings, and average proximity to the platform position using automated video tracking. All mice subsequently completed control visible platform trials to verify the absence of sensory or motor impairments. Between trials, animals were gently dried and maintained under a heat lamp to prevent hypothermia. To mitigate cumulative stress, tests were spaced by 1–3-day rest intervals [32, 33], with aversive tests (TST, FST) placed last. All procedures were performed between 9:00 AM and 5:00 PM by an experimenter blinded to group assignments, with separate researchers handling coding and data analysis.

### Immunofluorescence staining

Mouse brains were fixed in 4% paraformaldehyde (PFA) overnight, dehydrated gradient concentration of sucrose solution, and embedded in optimal cutting temperature compound (4583, Sakura Finetek, Torrance, CA, USA). Frozen coronal Sect. (30 mm thick) containing the hippocampus were obtained by using a freezing microtome (CM3050S, Leica, Wetzlar, Germany) and mounted on adhesion microscope slides (188105, Citotest, Jiangsu, China). Neuroectodermal (NE-4c) cells were fixed with 4% PFA for 15 min. The slices or cells were incubated for 1 h with 0.1% Triton X-100 in QuickBlock Blocking Buffer (P0235, Beyotime, Jiangsu, China), incubated with primary antibodies overnight at 4 °C, and incubated for 1 h at 37 °C with Alexa Fluor-conjugated secondary antibodies (Invitrogen). Hoechst (C1018, Beyotime) was used for nuclear staining. The primary antibodies included mouse anti-NEUN (ab104224; Abcam, Cambridge, MA, USA), rabbit anti-DCX (ab77450, Abcam), rat anti-GFAP (13–0300, Invitrogen, glial fibrillary acidic protein, astrocyte marker), mouse anti-NESTIN (ab11306, Abcam), rabbit anti-NESTIN (ab221660, Abcam). Alexa Fluor-conjugated secondary antibodies included goat anti-mouse IgG (H + L) highly cross-adsorbed secondary antibody, Alexa Fluor 555 (A-21424, Invitrogen), goat anti-rabbit IgG (H + L) crossadsorbed secondary antibody, Alexa Fluor 488 (A-11008, Invitrogen) and Alexa Fluor 555 (A-21428, Invitrogen), donkey anti-goat IgG (H + L) cross-adsorbed secondary antibody, Alexa Fluor 594 (A-21209, Invitrogen). Images were captured using a Leica TCS SP8 confocal microscope. All samples were processed using identical tissue fixation, dehydration, and embedding protocols, any subtle shrinkage would occur uniformly across all groups. For tissue processing and imaging, brain sections were numerically recoded by an independent researcher not involved in subsequent steps. The experimenter performing immunohistochemical staining was blinded to sample identity. Image acquisition and quantitative analysis were carried out by another researcher who was also unaware of group assignments.

### NE-4c cell culture and treatment

NE-4c cells (ZQ0275, ScienCell, Shanghai, China) were cultured in mouse NSC complete medium (J10001-4, ScienCell). The cells were seeded at 1 × 10^5^ cells/mL density into poly-D-lysine-coated 25 or 75 cm^2^ flasks. When the cells reached 90% confluence, they were harvested with trypsinization in 37 °C cell incubator for 1 min and then seeded in 6-, 24-, 96-well plates at 1 × 10^5^ cells/mL density according to different experimental requirements. NE-4c cells were treated by fructose (5 mmol/L) at about 50% confluence, then continued to cultivate until harvest.

### RNA sequence (RNA-seq)

The RNA of NE-4c cells induced by fructose was isolated with miRNeasy Micro Kit (1071023, Qiagen, Hilden, Germany) according to the manufacturer protocol. The RNA-Seq was performed performed, processed and analyzed with standard protocols at Majorbio Co., Ltd (Shanghai, China).

### Tunel

Chromosomal DNA damage was evaluated using the fluorescein Tunel detection kit (G1501, Servicebio, Wuhan, China). Tunel positive cells were detected according to the manufacturer instructions.

### Quantitative real-time PCR (qRT-PCR)

Trizol reagent (15596026, Invitrogen) was used to isolate total mRNA from mouse hippocampus and NE-4c cells, respectively. Reverse transcription was performed using the HiScript II select qRT supermix (R222-01, Vazyme, Jiangsu, China). qRT-PCR was performed on a qRT-PCR detection system (Bio-Rad Laboratories, Hercules, CA, USA) using SYBR Green (Q311-03, Vazyme) and gene specific primer sets. All primers (Suppl. Tables 1 and Table 2) were designed by us and synthesized in the Sangon Biotech Co. Ltd (Nanjing, China).

### Western blotting

Cells or tissues lysates were prepared in ice-cold RIPA buffer containing protease and phosphatase inhibitors. After quantifying protein concentration with a bicinchoninic acid protein assay kit (23005, Thermo Scientific, Waltham, MA, USA), equal aliquots were resolved by SDS-PAGE and electrophoretically transferred to PVDF membranes. Subsequently, the membranes were incubated in 5% skimmed milk, probed overnight at 4 °C with primary antibodies and then incubated with HRP-conjugated secondary antibodies. Primary antibodies included rabbit anti-GPX4 (ab125066, Abcam), rabbit anti-FTH1 (ab183781, Abcam), rabbit anti-SOD1 (ab308181, Abcam), rabbit anti-NRF2 (12721, CST, Massachusetts, USA), mouse anti-GAPDH (60004-1-1 g, protein-tech). The protein bands were detected by Tanon-5200 Chemiluminescence Imager (Tanon Science & Technology Co., Ltd., Shanghai, China). The density of bands was quantified using ImageJ (Version 1.50 b, National Institutes of Health, USA), normalized to internal reference protein and expressed as fold change relative to the control value.

### Lipid peroxidation detection

A lipid peroxidation kit (S0131, Beyotime) was used to test MDA content in NE-4c cells. Lipid peroxides react with thiobarbituricacid reagent at 100 °C for 15 min to produce a stable MDA-TBA adduct with a maximum absorption peak at 532 nm. Moreover, live-cell analysis reagent BODIPY 581/591 C11 (D3861, Invitrogen, WA, USA) was chosen to detect lipid peroxidation in NE-4c cells and mouse primary NSCs.

### Mitochondrial iron and reactive oxygen species (ROS) detection in NE-4c cells

mitochondrial iron and ROS levels were assessed in NE-4c cells using the mito-Fe^2+^ probe (M489, Dojindo, Beijing, China) and mito-ROS indicator (16052, AAT Bioquest, CA, USA), respectively. Cells were seeded in confocal dishes and subjected to the designated treatments. After treatment, cells were incubated with 5 µM mito-Fe^2+^ and 10 µM mito-ROS dye in serum-free medium at 37 °C for 30 min in the dark. Following staining, cells were washed twice with PBS and imaged immediately using a laser scanning confocal microscope.

### Statistics

All data was presented as mean ± standard error of mean (SEM). Two-tailed Student’s t test was carried out for two group comparisons. Data of three and more groups with single factor variance, were analyzed by one-way ANOVA for comparisons. *P* < 0.05 is statistically significant. The post hoc test performed after the one-way ANOVA is Bartlett’s test.

## Results

### High-fructose diet induces depressive-like behaviors and short-term memory impairment in mice

**Fig. 1:**
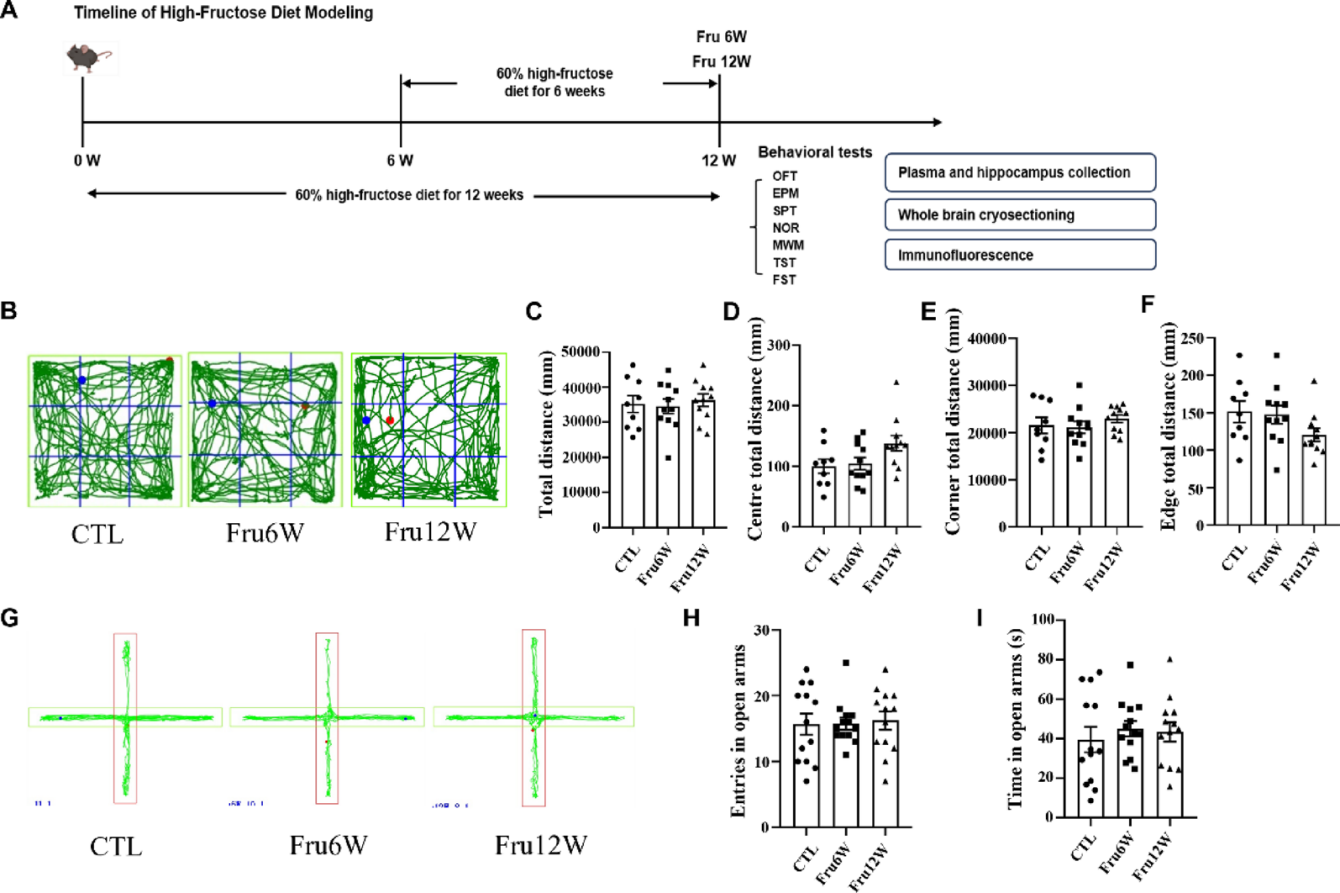
Effect of HFrD on anxiety-like behaviors in mice. (**A**) Experimental timeline of HFrD intervention and behavioral tests. (**B**) Representative movement traces from OFT. (**C**) Statistical results of the total distance in OFT. (**D**) Statistical results of the center distance in OFT. (**E**) Statistical results of the corner distance in OFT. (**F**) The statistical results of the edge distance in OFT. (**G**) Representative state diagram of the movement trajectory of EPM experimental animals, the green line in the figure is the movement trajectory, the red box is the open arm, and the rest is the closed arm. (**H**) Number of open arm entries in EPM. (**I**) Duration spent in open arms of EPM. CTL: control group, Fru6W: 6-week HFrD group, Fru12W: 12-week HFrD group. Data are expressed as Mean ± Sem, *P < 0.05, **P < 0.01, ***P < 0.001

To assess the impact of excessive fructose intake on emotional and memory functions, mice underwent a series of behavioral tests following 6- or 12-week of HFrD intervention (Fig. 1A). In the OFT, no significant differences were observed in total distance traveled or time spent in the center/peripheral zones between HFrD-fed and control mice (Fig. 1B-F). Similarly, the elevated plus maze test revealed comparable open arm entries and duration in both groups (Fig. 1G-I), suggesting that HFrD did not affect locomotor activity or anxiety-like behaviors.

**Fig. 2:**
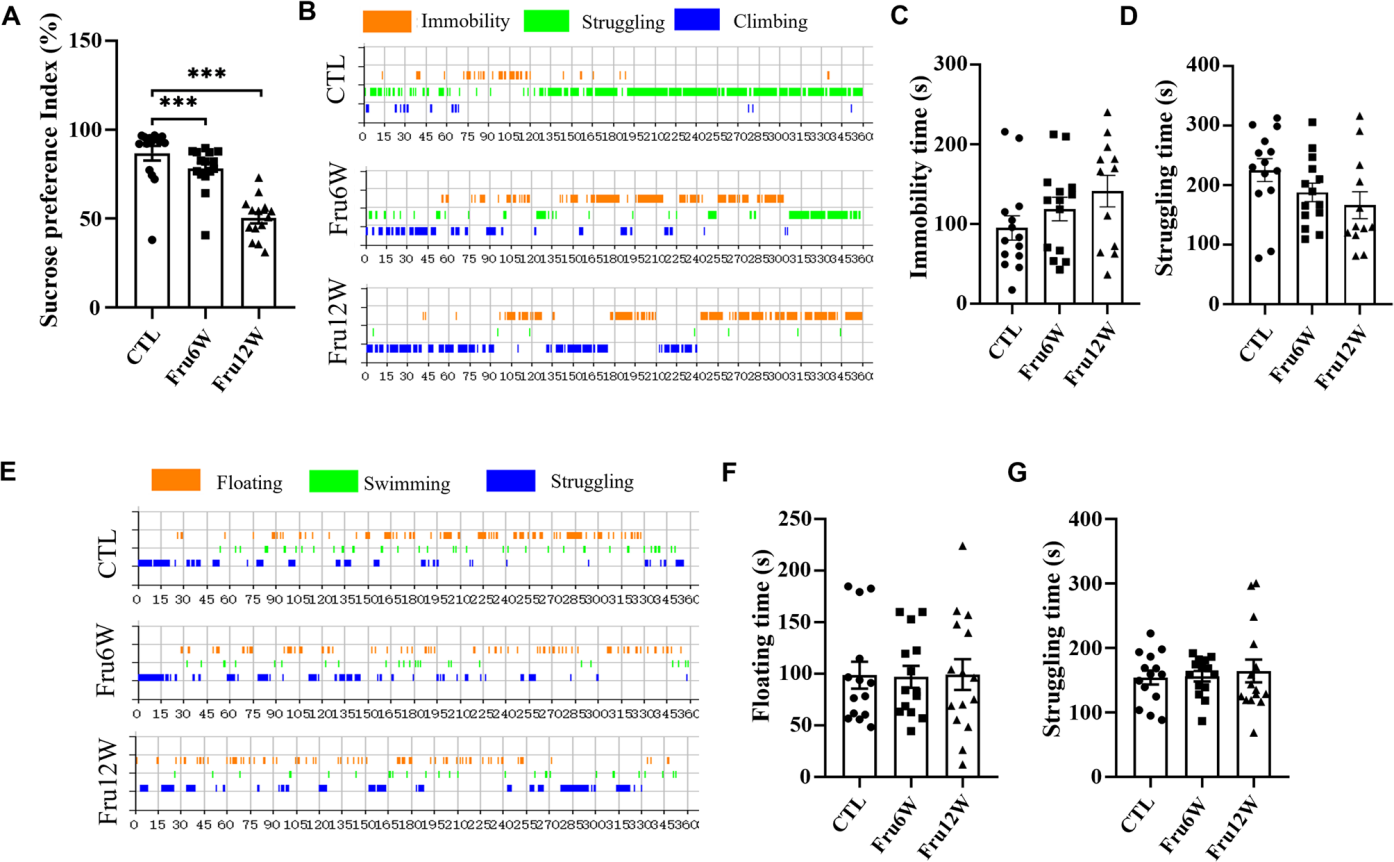
Effect of HFrD on depressive-like behaviors in mice. (**A**) Sucrose preference in SPT. (**B**) Representative state diagram was shown in TST. (**C**) The immobile time of mice was recorded in TST. (**D**) The struggling time of mice was recorded in TST. (**E**) Representative state diagram was shown in FST. (**F**) The floating time of mice was recorded in FST. (**G**) The struggling time of mice was recorded in FST. CTL: control group, Fru6W: 6-week HFrD group, Fru12W: 12-week HFrD group. Data are expressed as Mean ± Sem, *P < 0.05, **P < 0.01, ***P < 0.001

However, the SPT demonstrated a marked reduction in sucrose consumption in HFrD mice (Fig. 2A), suggesting anhedonia-like behavior. Moreover, the TST demonstrated a marginally non-significant reduction in struggling time in 12-week HFrD-fed mice (*p* = 0.0684), potentially indicating the onset of behavioral despair (Fig. 2B-D). In contrast, the FST showed no statistically significant alterations in either floating duration or active struggling time (Fig. 2E-G).

**Fig. 3:**
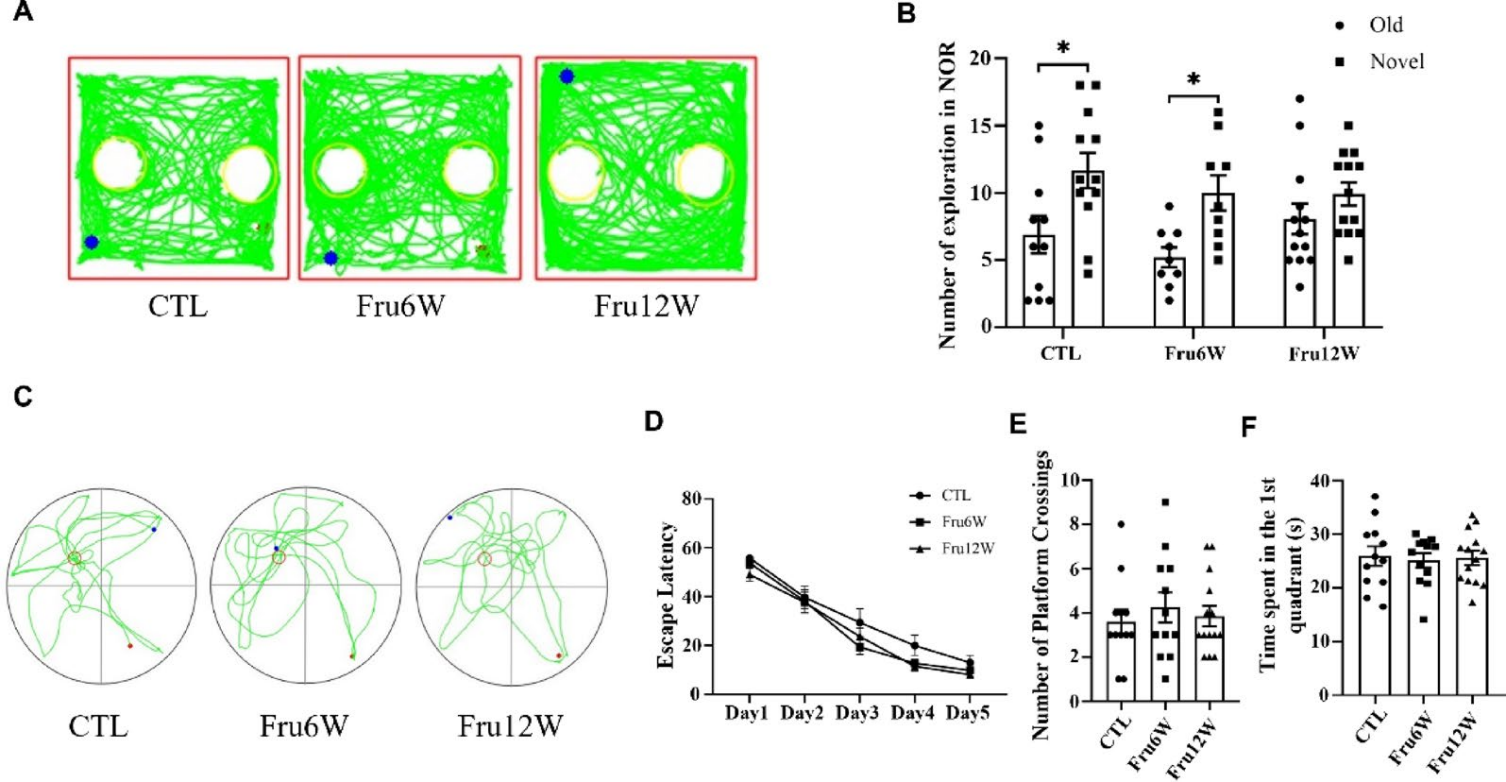
Effect of HFrD on short-term and spatial memory in mice. (**A**) Representative state diagram of NOR experiments. (**B**) The number of explorations in NOR experiment. (**C**) The escape latency of each group during the MWM experimental training period, and the shortening of the latency period represents memory formation. (**D**) Representative movement trajectories during the test session, with the green line indicating the animal locomotion path. (**E**) The number of platform crossings in mice during the test period. (**F**) Statistical chart of the residence time of mice in the first quadrant during the test period. CTL: control group, Fru6W: 6-week HFrD group, Fru12W: 12-week HFrD group. Data are expressed as Mean ± Sem, *P < 0.05, **P < 0.01, ***P < 0.001

The NOR test demonstrated significant impairment in short-term memory in 12-week HFrD mice, as indicated by a complete loss of novel object preference (Fig. 3A-B). In contrast, spatial learning and memory assessed by MWM showed no diet-induced differences, with comparable performance in escape latency during acquisition training and equivalent platform crossings and target quadrant preference during probe trials (Fig. 3C-F).

### High-Fructose diet impairs hippocampal neurogenesis

**Fig. 4:**
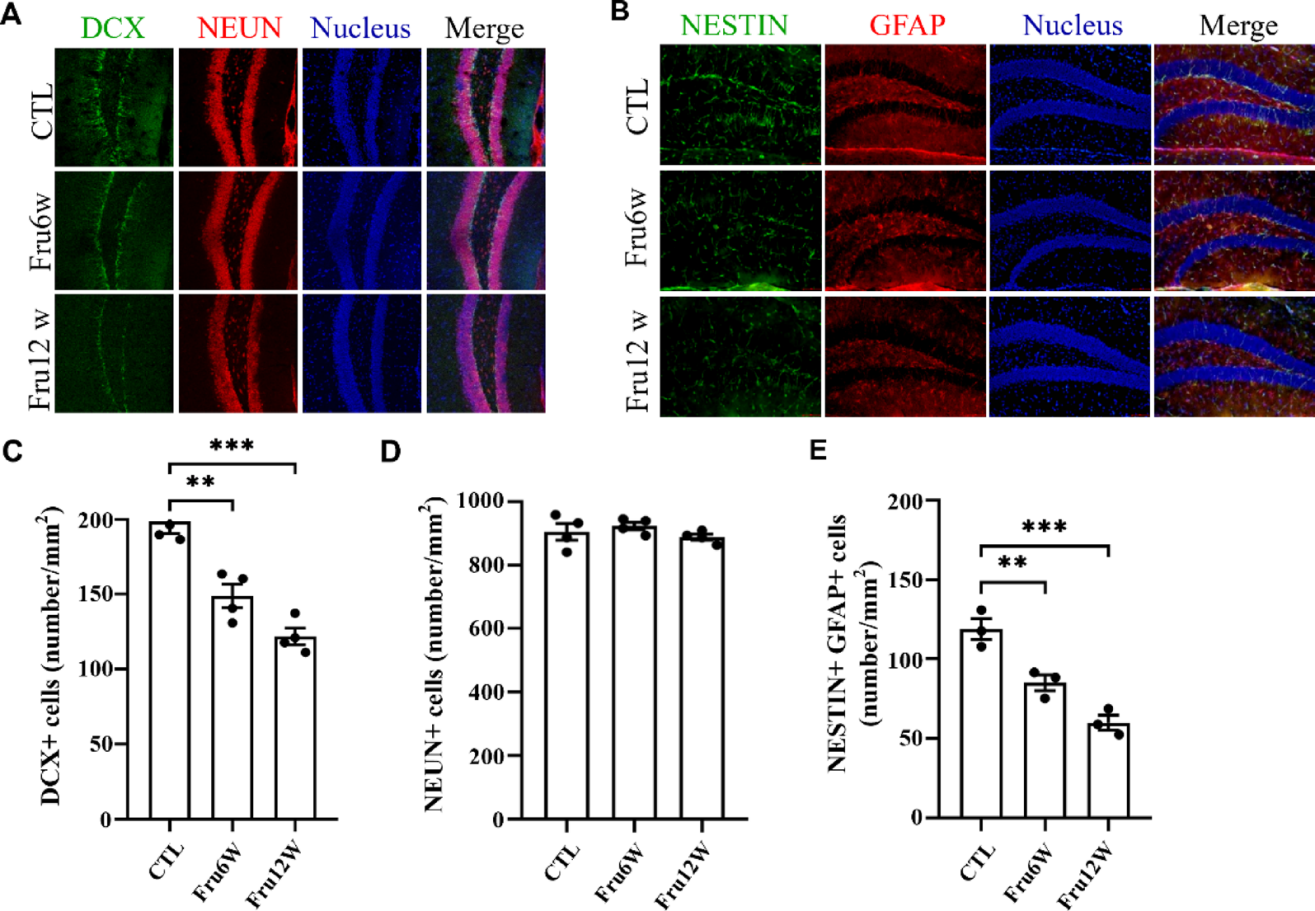
HFrD impairs hippocampal NSC pool neurogenesis in mice. (**A**) Staining using coronal section of mice, representative images labeled with DCX and NEUN were shown in mouse hippocampus. (**B**) Representative images of co-labeling with NESTIN and GFAP in the DG region of the mouse hippocampus. (**C**) The number of DCX-labeled newborn neurons. (**D**) The number of NEUN-labeled mature neurons was quantified in the mouse hippocampus. (**E**) The number of NSCs co-labeled with NESTIN and GFAP was quantified in the mouse hippocampus. CTL: control group, Fru6W: 6-week HFrD group, Fru12W: 12-week HFrD group. Data are expressed as Mean ± Sem, *P < 0.05, **P < 0.01, ***P < 0.001

To investigate the temporal effects of long-term HFrD on hippocampal neurogenesis, we systematically examined the dynamics of neurogenic activity at baseline (0-week), 6-week, and 12-week intervals post-HFrD initiation using immunofluorescence staining. The results showed comparable numbers of mature neurons across hippocampal subregions among groups, while significant reductions were observed in NSCs and immature neurons within the dentate gyrus after 6-week or 12-week HFrD induction (Fig. 4A-E; and Suppl. Figure 1 A-C). SPT score exhibited strong positive correlations with DCX count (*R* = 0.72, *P* < 0.01) and NSC count (*R* = 0.67, *P* < 0.01). The NOR discrimination index showed moderate to weak positive correlations with DCX count (*R* = 0.42, *P* < 0.01) and NSC count (*R* = 0.30, *P* < 0.05) (Suppl. Figure 2). These findings indicate that HFrD impairs hippocampal neurogenesis by affecting NSCs in the dentate gyrus.

### Transcriptomic profiling reveals fructose-induced disruption of energy metabolism in NSCs

**Fig. 5:**
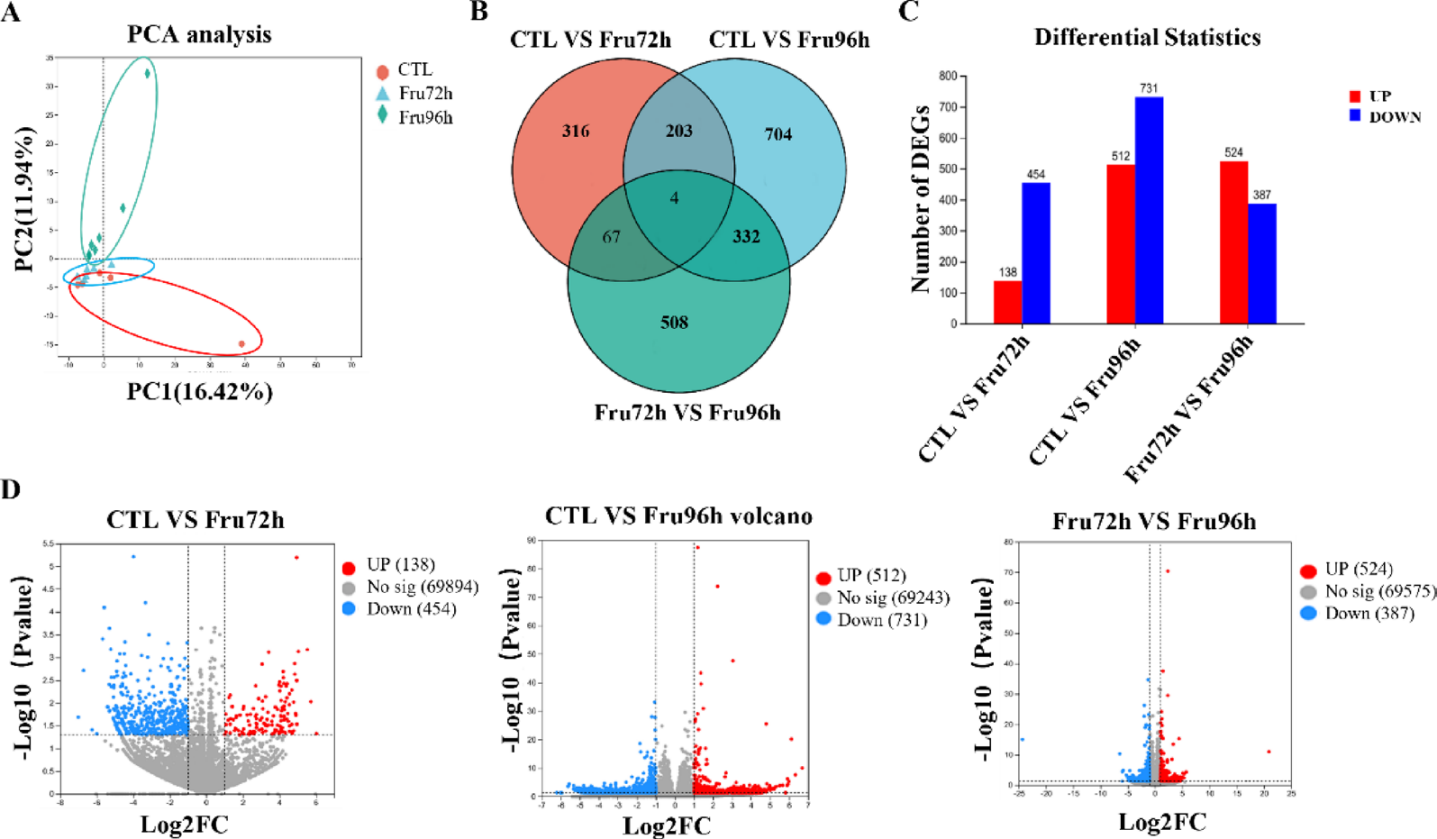
Results of DEGs analyzed by RNA-seq in NE-4c cells stimulated by high-fructose exposure for 72–96 h. (**A**) PCA of RNA-seq data showed distinct clustering among groups. (**B**) Venn diagram illustrated overlapping and unique DEGs across treatment groups. (**C**) Quantitative distribution of up- and down-regulated DEGs. (**D**) Volcano map of DEGs among CTL, Fru72 and Fru96 groups. Data are expressed as Mean ± Sem, *P < 0.05, **P < 0.01, ***P < 0.001

To assess the metabolic effects of high-fructose exposure on NSCs, we performed RNA sequencing (RNA-seq) on NE-4 C cells treated with fructose for 72–96 h. Principal component analysis (PCA) demonstrated a clear separation between the fructose-treated groups (Fru72h and Fru96h) and the control (CTL) group along the first two principal components (Fig. 5A). Venn analysis identified 316, 704, and 508 uniquely differentially expressed genes in the Fru72h vs. CTL, Fru96h vs. CTL, and Fru72h vs. Fru96h comparisons, respectively (Fig. 5B). Differentially expressed genes (DEGs) analysis identified 512 upregulated and 731 downregulated genes in 96 h fructose-treated cells compared to CTL (Fig. 5C-D).

**Fig. 6:**
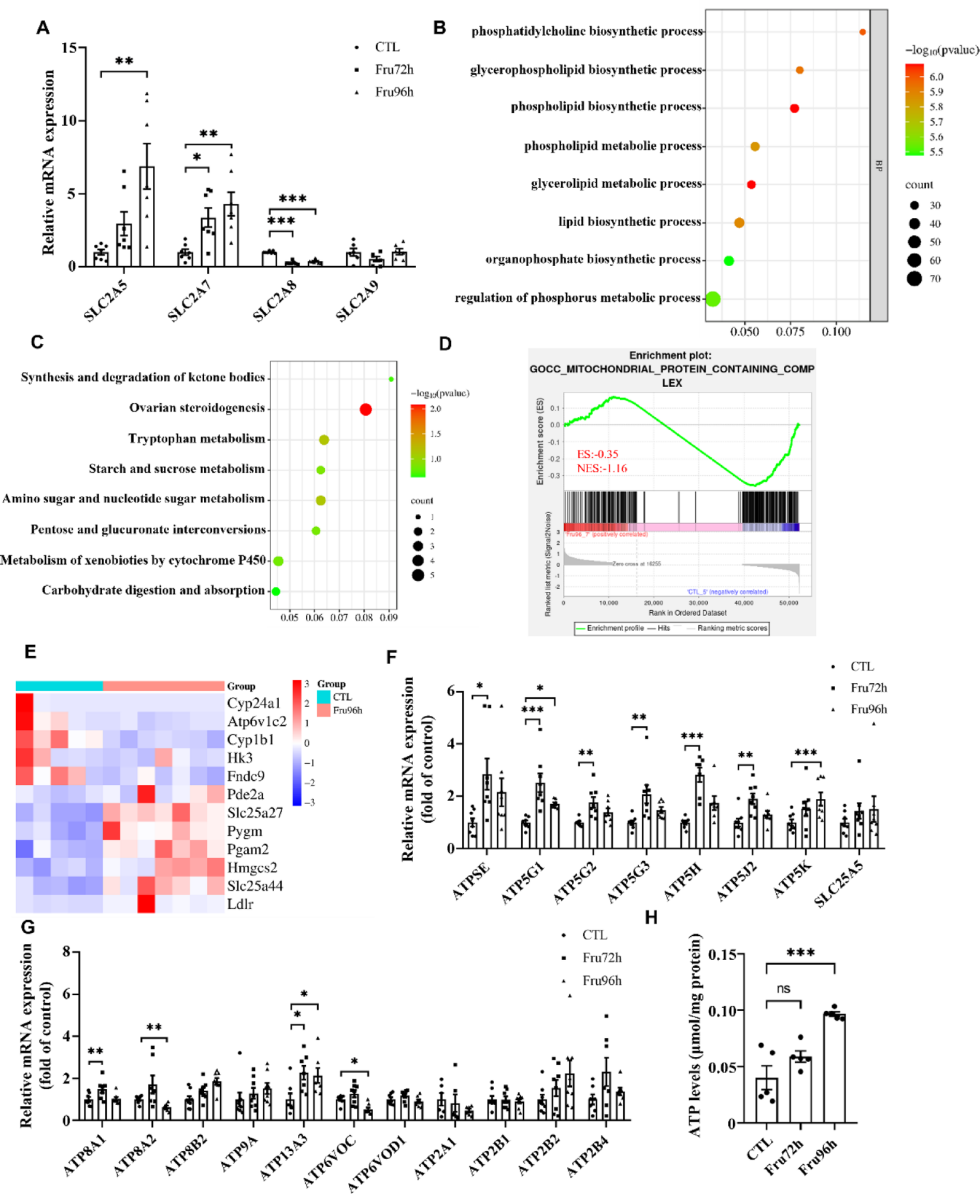
High fructose disrupted the expression of genes related to energy metabolism in NE-4c cells. (**A**) qPCR analysis of mRNA expression levels of fructose transporter-related genes in NE-4c cells. (**B**) GO analysis enrichment map of DEGs between CTL and Fru96 group. (**C**) KEGG analysis enrichment map of DEGs between CTL and Fru96 group. (**D**) GSEA analysis enrichment map of DEGs between CTL and Fru96 group. (**E**) Heatmaps of DEGs between CTL and Fru96 group. (**F**) qPCR analysis of the mRNA expression levels of genes related to ATP synthase in NE-4c cells (*n* = 6). (**G**) qPCR analysis of the mRNA expression levels of genes related to lipid metabolism, ion transport, and calcium ion transport in NE-4c cells (*n* = 6). (**H**) ATP content in NE-4c cells (*n* = 5). Data are expressed as Mean ± Sem, *P < 0.05, **P < 0.01, ***P < 0.001

Fructose exposure (72/96 h) significantly altered the mRNA expression of fructose transporters (SLC2A5, SLC2A7 and SLC2A8) in NE-4 C cells (Fig. 6A). RNA-seq further demonstrated that DEGs were enriched in biological processes related to lipid metabolism, including “phosphatidylcholine biosynthetic process,” “phosphatidylcholine metabolic process,” “lipid biosynctic process” and “glycerophospholipid biosynthetic process” (GO analysis, Fig. 6B). KEGG pathway analysis revealed that DEGs were associated with energy metabolism pathways, such as “starch and sucrose metabolism,” “carbohydrate digestion and absorption,” and “amino sugar and nucleotide sugar metabolism” (Fig. 6C).

Mitochondria serve as pivotal organelles that orchestrate cellular metabolism and energy production to critically regulate cell survival and death. Gene Set Enrichment Analysis (GSEA) using MSigDB and MitoCarta3.0 databases demonstrated that fructose-mediated NSC decrease was linked to “mitochondrial protein complex pathways” (Fig. 6D). Heatmap visualization highlighted key DEGs (Fig. 6E), which are involved in critical mitochondrial functions, including energy metabolism, protein synthesis, and cellular respiration. Notably, fructose exposure increased intracellular ATP levels in NE-4 C cells, accompanied by elevated mRNA expression of ATP synthase-related enzymes (ATP5E, ATP5G1, ATP5G2, ATP5G3, ATP5H, ATP5J2, ATP5K), lipid metabolism-related genes (ATP8A1, SYP8A2), ion transport-related genes (ATP13A3, ATP6V0C) but unchanged expression of the ATP transporter SLC25A5 and calcium/sodium ATPase genes (ATP2A1, ATP2B1, ATP8B2, ATP2B4) (Fig. 6F-H). These findings suggest that fructose enhances ATP synthesis without altering ATP transport capacity, leading to ATP accumulation and subsequent metabolic dysfunction.

### High-fructose diet promotes proliferation of NE-4c Cells In Vitro

**Fig. 7:**
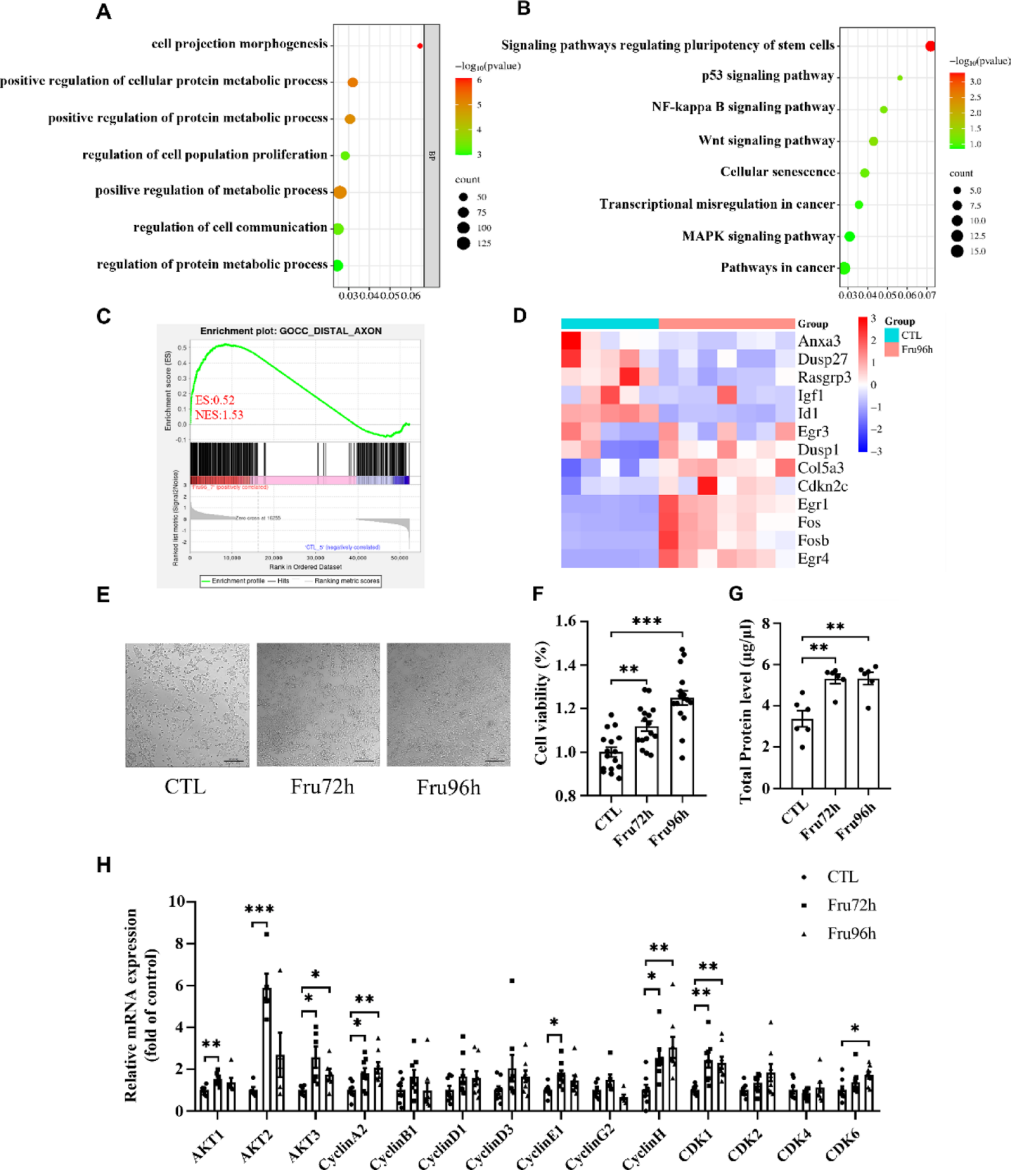
High fructose promotes the NSC proliferation.(**A**) GO analysis enrichment map of DEGs between CTL and Fru96 group in NE-4c cells. (**B**) KEGG analysis enrichment map of DEGs between CTL and Fru96 group in NE-4c cells. (**C**) GSEA analysis enrichment map of DEGs between CTL and Fru96 group in NE-4c cells. (**D**) Heat map of genes associated with proliferation in DEGs between CTL and Fru96 group in NE-4c cells. (**E**) Bright-field images of NE-4c cells in CTL, Fru72 and Fru96 groups (Scale bar: 50 μm). (**F**) Effect of different administration durations on the viability of NE-4c cells (n = 6). (**G**) NE-4c cell protein concentration determination in CTL, Fru72 and Fru96 groups (n = 6). (**H**) qPCR detection of mRNA expression levels of genes related to NE-4c cell proliferation (n = 6). Data are expressed as Mean ± Sem, *P < 0.05, **P < 0.01, ***P< 0.001

To determine the impact of fructose-induced metabolic dysregulation on NSC growth, we profiled DEGs between CTL and Fru96h groups. GO enrichment analysis demonstrated significant alterations in biological processes related to “regulation of cell population proliferation” (Fig. 7 A). KEGG pathway analysis further identified significant enrichment in key pathways governing NSC fate, including the MAPK and Wnt signaling pathways, both of which are critically involved in the regulation of NSC proliferation and differentiation (Fig. 7B). GSEA further localized fructose-mediated proliferative effects to the “distal axon” (Fig. 7 C). Notably, key proliferation-related genes (Egr1, Fos, Fosb, Egr4) were significantly upregulated in Fru96h-treated cells (Fig. 7D).

Microscopic examination revealed an increase in NE-4c cell density under fructose exposure (Fig. 7E). In vitro functional assays, including cell viability and total protein concentration measurements, further validated fructose-enhanced proliferative capacity (Fig. 7G-H). Additionally, mRNA levels of cell cycle regulators (CyclinA2, CyclinE1, CyclinH, CDK1) were markedly elevated in fructose-exposed NE-4c cells (Fig. 7 F). The observed in vitro proliferative effect contrasts with in vivo NSC depletion, suggesting that chronic fructose exposure may exhaust NSC reservoirs through dysregulated expansion. This aligns with previous reports by Xu et al. [18]. linking hyperactivation to long-term NSC pool depletion

### High-fructose exposure induces apoptosis in NE-4c cells

**Fig. 8:**
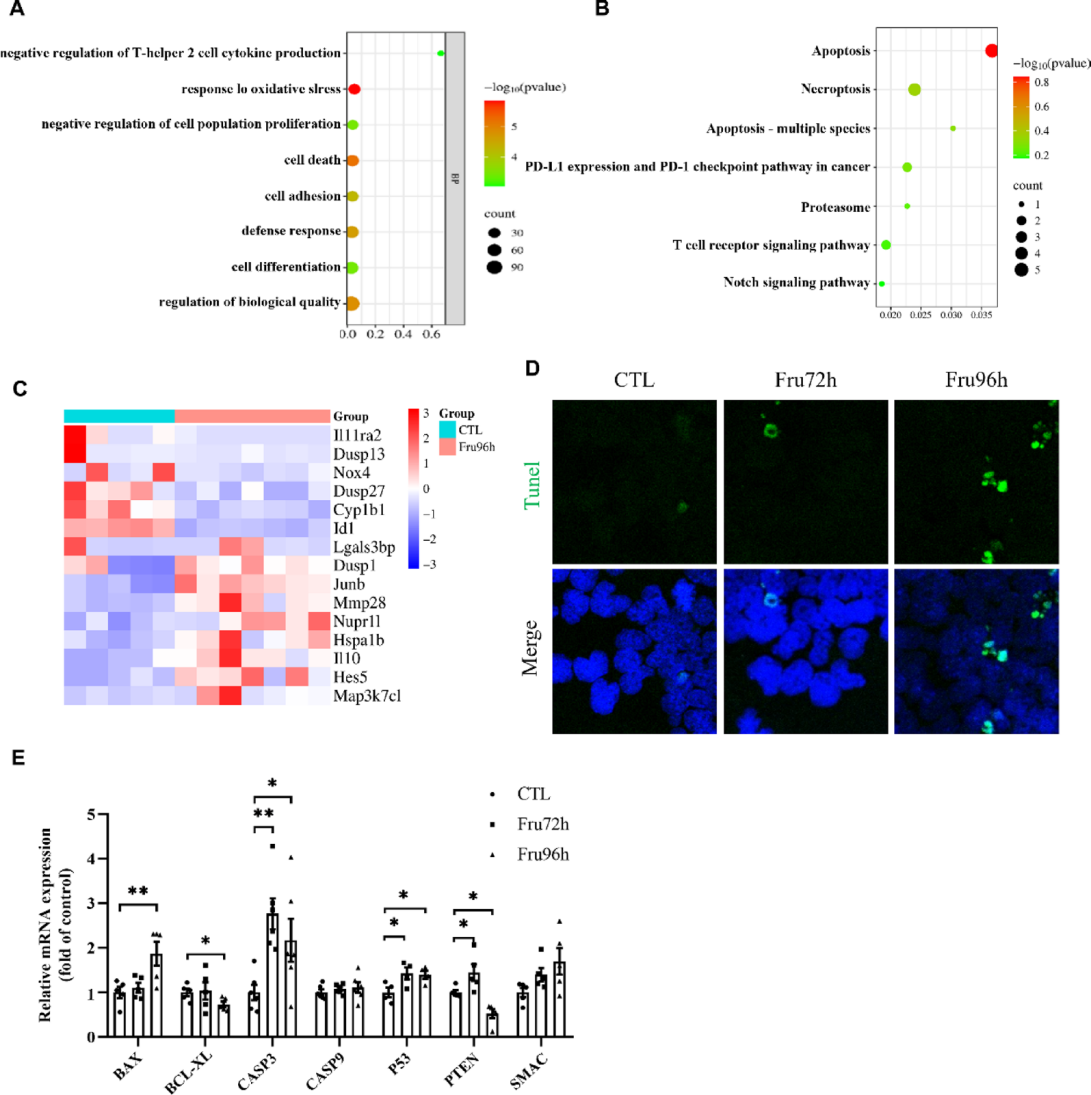
High fructose promotes NSC apoptosis. (**A**) GO enrichment analysis of DEGs between CTL and Fru96 group in NE-4c cells. (**B**) KEGG enrichment analysis of DEGs between CTL and Fru96 group in NE-4c cells. (**C**) Heat map of apoptosis-related DEGs between CTL and Fru96 group in NE-4c cells. (**D**) Tunel staining results of NE-4c cells in CTL, Fru72 and Fru96 groups. (**E**) qPCR analysis of mRNA expression levels of apoptosis-related genes in CTL, Fru72 and Fru96 groups. Data are expressed as Mean ± Sem, *P< 0.05, **P < 0.01, ***P < 0.001

RNA-seq further revealed that high-fructose exposure concomitantly regulated NSC death pathways. Apoptosis is a programmed cell death process. Our analysis identified key apoptosis-related genes through integrated GO and KEGG pathway analyses, revealing a significant enrichment of DEGs in “cell death” and “apoptosis” processes (Fig. 8A-B). Heatmap visualization highlighted core apoptotic DEGs (Fig. 8C).

To validate these findings, we performed Tunel staining and observed a marked increase in fluorescently-labeled apoptotic cells in Fru96h group compared to CTL under fluorescence microscopy (Fig. 8D). Consistent with this, quantitative PCR analysis revealed significant upregulation of pro-apoptotic regulators (BAX, Caspase 3, and P53) and downregulation of anti-apoptotic factor (BCL-XL) at the mRNA level in fructose-exposed NE-4 C cells (Fig. 8E). These results collectively demonstrate that prolonged fructose exposure promotes apoptotic cell death in NSCs.

### High-fructose exposure induces ferroptosis in NE-4c cells

**Fig. 9:**
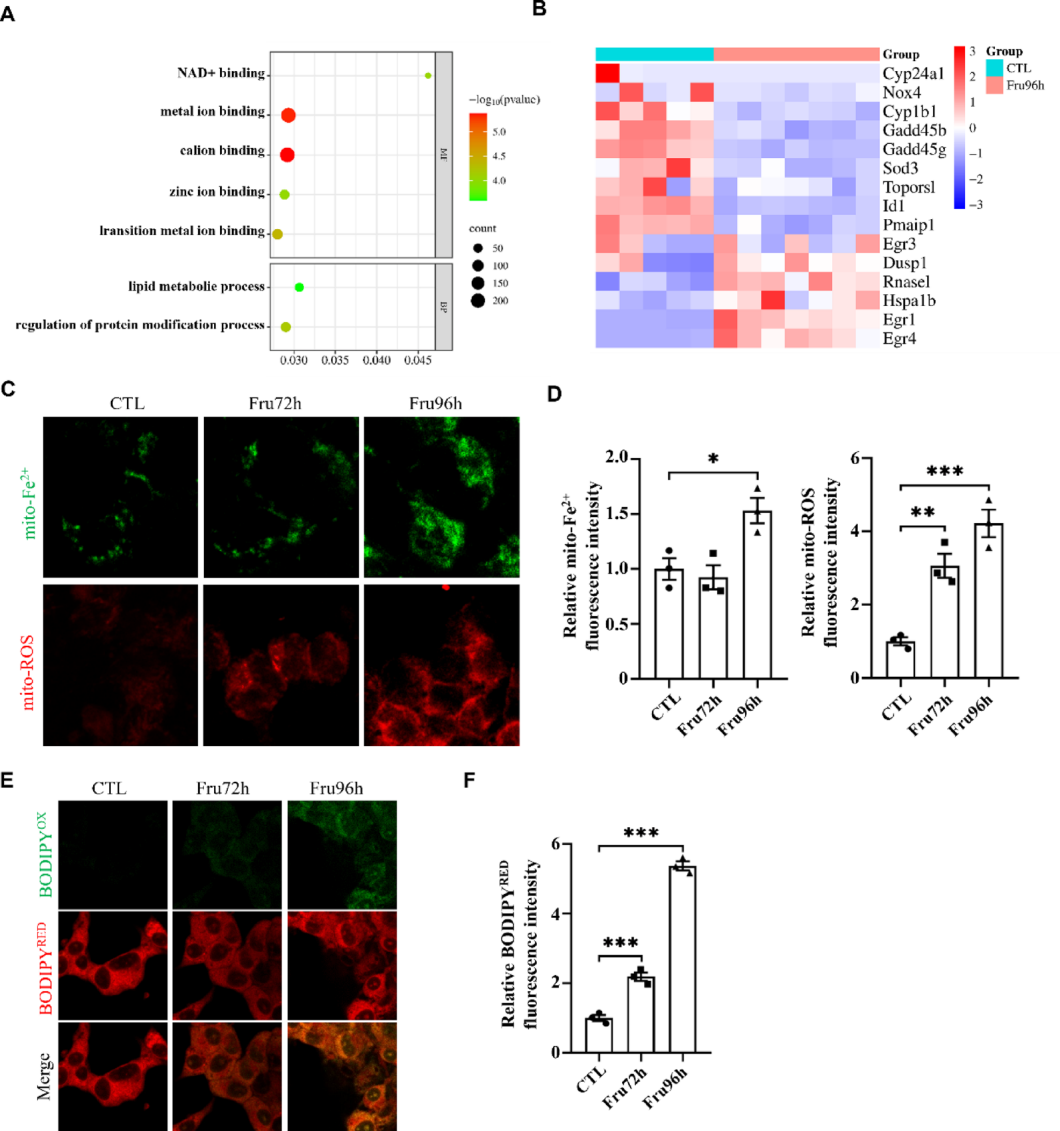
High fructose promotes NSC ferroptosis. (**A**) GO enrichment analysis of DEGs between CTL and Fru96 group in NE-4c cells. (**B**) Heatmap of ferroptosis-related DEGs between CTL and Fru96 group in NE-4c cells. (**C**) Representative images of mito-Fe^2+^ and mito-ROS levels in high-fructose-administered NE-4c cells. (**D**) Statistical results of mito-Fe^2+^ and mito-ROS concentration in NE-4c mitochondria. (**E**) Representative images of NE-4c intracellular lipid peroxidation levels. BODIPY^OX^ is used to label oxidized lipids within cells, and BODIPY^RED^ is used to label total lipids within cells. (**F**) Fluorescence intensity of lipid peroxidation in NE-4c cells. Data are expressed as Mean ± Sem, *P < 0.05, **P < 0.01, ***P< 0.001

Ferroptosis, an iron-dependent form of regulated cell death, is characterized by excessive intracellular iron accumulation and lipid peroxidation. GO enrichment analysis demonstrated that these DEGs were predominantly involved in molecular functions related to “metal ion binding”, with significant enrichment in cellular “lipid metabolic processes” (Fig. 9A). Heatmap analysis revealed distinct expression patterns of ferroptosis-related DEGs (Fig. 9B). Elevated mitochondrial Fe²⁺ and ROS levels were found in Fru96h group versus CTL group (Fig. 9C, D). Notably, BODIPY™ 581/591 C11 oxidation (BODIPYox) assays confirmed substantially increased lipid peroxidation in Fru96h groups (Fig. 9E).

**Fig. 10:**
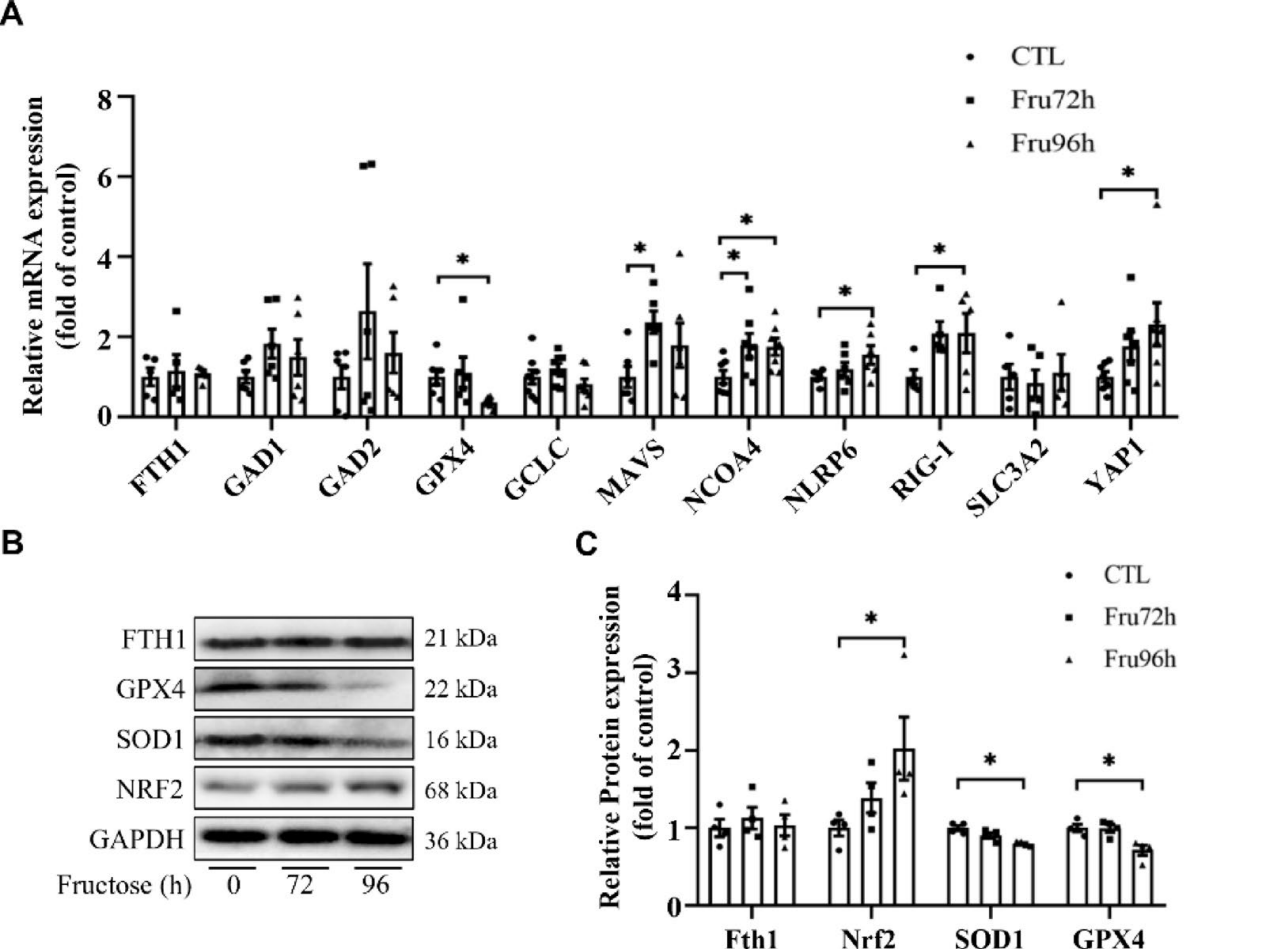
High fructose promotes NSC ferroptosis by inhibiting GPX4 and SOD1 expression. (**A**) qPCR detection of mRNA expression levels of ferroptosis-related genes in NE-4c cells. (**B**) Western blotting was used to detect ferroptosis-related protein expression in NE-4c cells. (**C**) Relative protein expression level was shown. Data are expressed as Mean ± Sem, *P < 0.05, **P < 0.01, ***P< 0.001

Quantitative PCR and immunoblotting analyses identified significant alterations in ferroptosis-related genes. Following 96 h fructose stimulation, qPCR analysis revealed a significant reduction in GPX4 mRNA levels, accompanied by elevated expression of NCOA4, RIG-1, and YAP1 transcripts (Fig. 10A). Western blotting confirmed these findings at the protein level, demonstrating decreased GPX4 and SOD1 expression alongside upregulated NRF2, while FTH1 protein abundance remained unaltered (Fig. 10B, C).

## Discussion

**Fig. 11:**
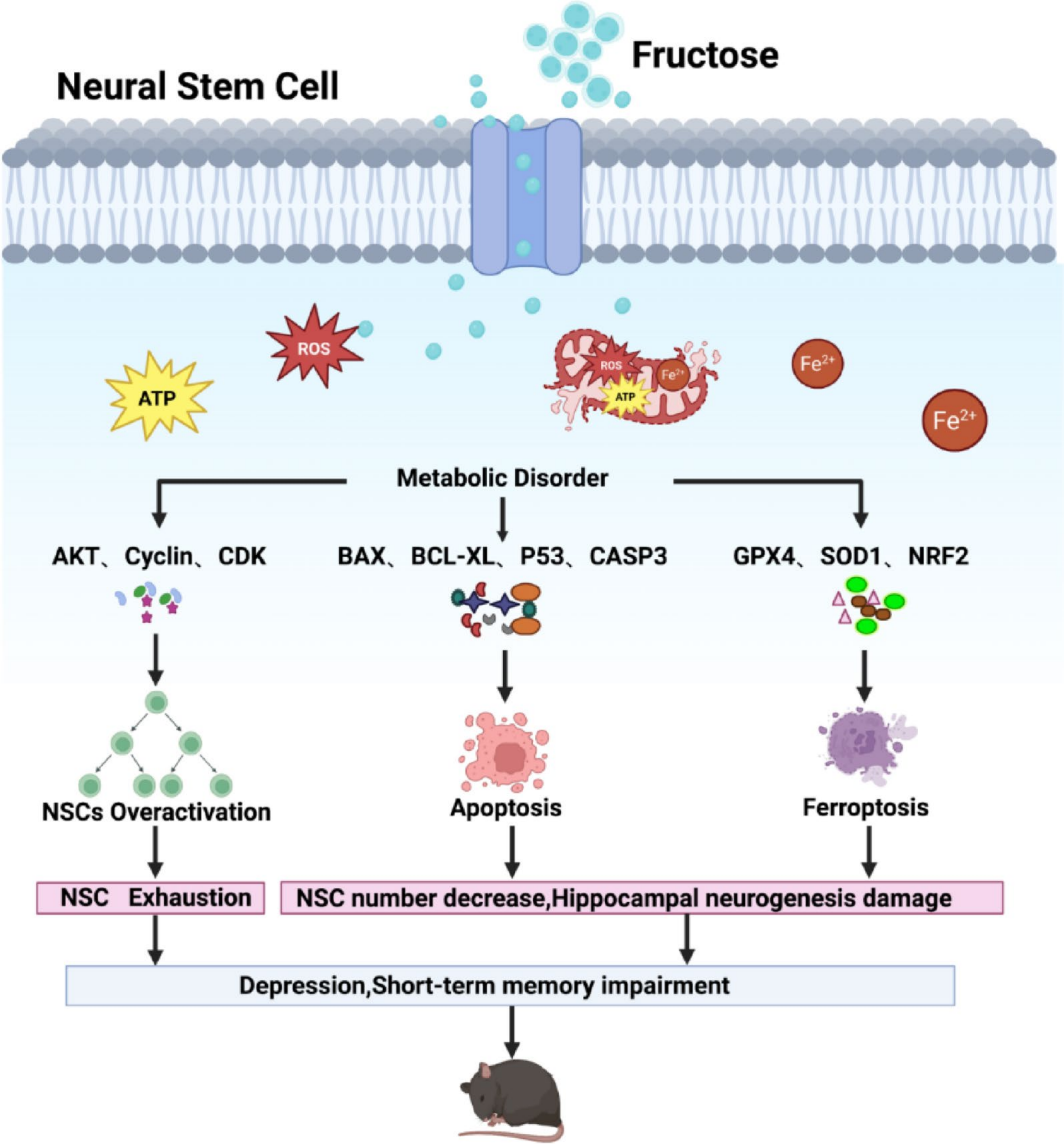
The mechanism by which fructose decreases hippocampal NSC number and leads to depression and short-term memory impairment in mice. Fructose enters NSCs through fructose transporter, triggering metabolic disorder with increased ATP and mito-Fe^2+^ and mito-ROS. Metabolism disorder leads to the depletion of the hippocampal NSC pool in mice by causing abnormal proliferation, apoptosis, and ferroptosis. The depletion of the NSC pool further results in depressive-like behavior and short-term memory impairment in mice

Our findings reveal that HFrD induces depressive-like behaviors and short-term memory impairment in mice, likely mediated by metabolic dysregulation in NSCs. The observed neurobehavioral deficits correlate with HFrD-induced NSC dysfunction, characterized by concurrent hyperactivation of proliferative pathways and elevated apoptotic/ferroptotic cell death. Notably, metabolic disturbances such as disrupted ATP homeostasis, iron accumulation, and lipid peroxidation underlie these pathological NSC responses. These results establish a direct link between fructose-mediated NSC metabolic stress and hippocampal-dependent cognitive-emotional deficits, offering mechanistic insights into diet-induced neuropsychiatric disorders (Fig. 11).

Fructose is a natural monosaccharide that is widely used in modern society. In recent years, it has been found that excessive intake of fructose can induce neuropsychiatric diseases. In this study, significant decrease was observed in the groups fed with a HFrD for 6 or 12 weeks compared with control group in the SPT. These findings suggest that a high-fructose diet affects the taste preference, resulting in anhedonia of mice, which is consistent with the results of many existing studies. Moreover, in the TST, the behavioral change of the mice in the group fed with high fructose for 12 weeks, characterized by a reduced struggling time (*p* = 0.0684), suggests that the mice may exhibit behavioral despair. However, there were no significant behavioral changes observed in the mice on a high-fructose diet during the FST. These results show some discrepancies compared with existing studies [34, 35]. For examples, Harrell CS et al. found that periadolescent rather than adult rats given a HFrD for 8–10 weeks showed significantly less time struggling in FST than standard chow fed rats [34]. Chakraborti et al. found that high fructose corn syrup-moderate fat diet for 16 weeks displayed enhanced anxiogenesis, increased behavioral despair, and impaired social interactions in C57BL/6 mice [35]. Yankelevitch-Yahav et al. demonstrated that the FST used for assessing or inducing depressive-like behaviors in rodents, exhibits limited sensitivity due to significant variability influenced by intrinsic factors (e.g., individual animal state) and extrinsic experimental conditions [36]. Therefore, the absence of significant despair-like depressive behaviors in our mice after 12-week HFrD may result from either the late diet initiation (at 8-week-old) or the stronger fructose tolerance of C57BL/6 mice compared to rat models.

The observed reduction in sucrose preference strongly indicates anhedonia, a core symptom of depression, reflecting deficits in reward processing. The trend toward increased immobility in the TST *(p* = 0.0684) after 12 weeks HFrD suggests a potential emergence of behavioral despair, which may become significant with prolonged exposure or greater sample size. In contrast, the absence of a significant effect in the FST may reflect differences in test sensitivity, motivational requirements, or underlying neural substrates. The FST is known to involve both serotonergic and noradrenergic mechanisms [37, 38], while the TST and anhedonia in the SPT may be more closely linked to dopaminergic and mesolimbic dysfunction [39–41], which could be preferentially affected by HFrD. Furthermore, the physical activity required in the FST may engage distinct physiological pathways less susceptible to metabolic disruption induced by fructose. The NOR test evaluates spontaneous discrimination between novel and familiar objects without reinforcement [42]. It relies on a highly specific neural circuit with minimal redundancy, critically involving the hippocampus [43], and especially the dentate gyrus, which supports pattern separation of similar sensory inputs [44]. Due to this limited redundancy, impaired neurogenesis in the dentate gyrus readily impairs NOR performance, as no alternative pathways compensate for deficits in object-specific encoding. Moreover, the absence of external reward or punishment in NOR [45] removes potential motivational influences that could mask mild memory deficits, allowing such impairments to directly manifest as reduced discrimination. In contrast, the MWM is a reinforced task where motivation is driven by escape from water. It engages broader and more redundant neural circuits. Spatial memory in the MWM can be supported by alternative pathways, such as those involving anterior thalamic–hippocampal connections [46]. Repeated training over multiple days promotes gradual stabilization of spatial memory through de novo protein synthesis, enabling compensation for mild synaptic impairments. Furthermore, reward contingency modulates medial septal–hippocampal activity, including theta oscillations and place cell firing [47], which enhances the resilience of the spatial navigation circuit.

Current dietary guidelines, such as those from the WHO and the UK Scientific Advisory Committee on Nutrition (SACN), recommend that sugar intake be limited to less than 10% and 5% of total energy intake, respectively [48, 49]. Actual sugar consumption in European populations varies between 7% and 25%, which remains considerably lower than the 60% fructose diet used in this study [50]. Although this level of fructose exceeds typical human intake, high-fructose diets are well established in nutritional neuroscience for investigating mechanistic links between excessive fructose consumption and central nervous system pathologies [5, 51–53]. Our results highlight the neurocognitive risks associated with chronically elevated fructose intake, even at moderate levels, and identify key metabolic pathways that may underlie its pathogenic effects. Thus, while not representative of common consumption patterns, this model serves as a validated tool for uncovering mechanisms relevant across a range of exposures, offering important insights into the neuropathology associated with high sugar diets and ultra-processed foods. The in vitro fructose concentration (5 mM) used in this study exceeds typical systemic levels but was selected to model the sustained metabolic stress induced by chronic high fructose intake. This treatment reliably induced apoptosis and ferroptosis in NE-4 C cells via the p53/Wnt pathway, thereby providing a robust and relevant cellular model for investigating the neurotoxic effects of long-term dietary fructose exposure.

Reversible switch between activated and quiescent status maintains the long-lived pool of NSCs in adult brain, which not only confers protection from damage but also prevents irreversible depletion of NSC pool [54]. Quiescent NSCs rely primarily on glycolysis to maintain their energy supply, while activated NSCs and their differentiated neuronal progeny are characterized by increased mitochondrial oxidative phosphorylation (OXPHOS) [55]. The NSC proliferation in the murine hippocampus represents a double-edged sword. Physiological proliferation maintains NSC renewal, thereby preserving a normative decline rate in the NSC pool [7]. Conversely, excessive aberrant proliferation may trap NSCs in a persistently activated state, preventing their return to quiescence and ultimately depleting the NSC reservoir [22]. Xu et al. demonstrated that Arg-II deficiency leads to NSC excessive proliferation, thereby resulting in NSC pool depletion over time. Corroborating these findings, the number of total and proliferating NSCs, as well as their progeny, was significantly reduced in Arg-II KO mice at 6 months of age [22]. The decrease in Id4 caused by knocking down Mysm1 account for the overactivation and decreased glial formation of NSCs [23]. Here, we found fructose diet is also important for NSC fate decision. High fructose stimulation in vitro promotes the NSC proliferation (Fig. 7). In vivo, NSC number was significantly decreased in mice fed a HFrD for 6 and 12 weeks (Fig. 4). Consequently, HFrD may deplete the hippocampal NSC pool through excessive activation of NSCs in mice. Of course, this requires further validation through comprehensive temporal monitoring of NSC quantity and proliferative capacity in mice following HFrD exposure—a limitation of the current study.

The contrasting responses of NSCs and mature neurons to HFrD exposure reveal a previously unrecognized duality in cell death mechanisms within the hippocampal niche. While existing studies primarily demonstrate elevated neuronal fructose uptake leads to intracellular fructose accumulation, subsequently inducing neuronal apoptosis in rats [56]. Our findings establish that NSCs exhibit delayed but progressive vulnerability characterized by concurrent activation of apoptotic and ferroptotic pathways following chronic HFrD exposure (12-week). This temporal and mechanistic divergence—where neurons succumb to enhanced GLUT5 expression [56] whereas NSCs accumulate mitochondrial iron overload and GPX4 suppression—may reflect fundamental differences in metabolic programming between these cell populations. Notably, the observed ferroptosis in NSCs parallels Warburg-effect-driven iron toxicity in cancer models [57], suggesting NSC-specific metabolic fragility in maintaining redox homeostasis during prolonged fructose stress. The requirement for dual death pathway inhibition (targeting both BCL-2 family proteins and lipid peroxidation) to preserve NSC pools underscores the complexity of dietary neurotoxicity and highlights the need for temporally stratified interventions in metabolic disorders. Our observation of concurrent ferroptosis and apoptosis in fructose-insulted NE-4 C neural cells aligns with the reported ability of liproxstatin-1 to concurrently attenuate ferroptosis, apoptosis, pyroptosis, and necroptosis in a mouse model of metabolic dysfunction-associated fatty liver disease [58]. This concordance suggests that fructose neurotoxicity is likely mediated by the coordinated activation of parallel cell death pathways, rather than through an isolated mechanism. Consequently, therapeutic strategies using a combination of ferroptosis and apoptosis inhibitors may demonstrate superior efficacy compared to monotherapeutic approaches targeting a single pathway.

Our findings implicate HFrD-induced metabolic stress in hippocampal NSCs as a contributor to cognitive decline. However, we recognize that additional mechanisms may participate in fructose-associated memory impairment. It is plausible that HFrD disrupts hippocampal functional plasticity, including neurogenic and synaptic processes. For example, ergothioneine (ERGO), dependent on OCTN1-mediated uptake, promotes neuronal differentiation of NPCs and enhances cognitive function via TrkB activation and synaptogenesis [59–61]. Conversely, HFrD may suppress these plasticity-promoting pathways, such as TrkB signaling and mTOR-dependent spine maturation, through sustained metabolic and oxidative stress. Thus, hippocampal dysfunction likely arises from both NSC-intrinsic metabolic deficits and impaired circuit-level plasticity, warranting further investigation into these complementary mechanisms.

## Conclusions

This study systematically deciphers the mechanisms by which HFrD impairs neural NSC homeostasis and hippocampal function. We demonstrate that HFrD induces metabolic disorder in NSCs, characterized by dysregulation of energy production and suppression of ferroptosis defense systems. These alterations trigger a dual cell death response—combining apoptotic and ferroptotic pathways—that depletes the NSC pool and compromises adult hippocampal neurogenesis. Crucially, our multi-modal approach integrating behavioral, histological, and omics analyses establishes a direct causal chain from HFrD-mediated NSC loss to the emergence of depressive-like phenotypes and short-term memory deficits.

## Supplementary Information

Supplementary Material 1.

## Data Availability

All data can be obtained from corresponding authors at any time upon reasonable request.
