# Supplementary material for: High-fructose diet induces depressive-like behaviors and short-term memory deficits through hippocampal neurogenesis impairment via neural stem cell dysfunction
Source: Nutr Metab (Lond). 2025 Dec 2;22:149. doi: 10.1186/s12986-025-01043-7 (PMC12673773; doi:10.1186/s12986-025-01043-7)
Supplement: Supplementary file 1 — Supplementary Material 1. [file 12986_2025_1043_MOESM1_ESM.docx]

Appendix A. Supplementary data for

High-fructose diet induces depressive-like behaviors and short-term memory deficits through hippocampal neurogenesis impairment via neural stem cell dysfunction

Qiaona Wang ^b^, Yongfa Wang ^b,c^, Yuefeng Hu ^b,c^, Pengfei Xie ^b,c^, Fan Li ^b,c^, Ruoyu Mu ^a^, Zhenjie Feng ^c^, Feng Zhou ^b,**^, Chuanfeng Tang ^a,*^

a State Key Laboratory of Technologies for Chinese Medicine Pharmaceutical Process Control and Intelligent Manufacture, Nanjing University of Chinese Medicine, Nanjing, 210023, People's Republic of China.

b School of Food Science, Nanjing Xiaozhuang University, Nanjing 211171, People's Republic of China.

c School of Food Science and Pharmaceutical Engineering, Nanjing Normal University, Nanjing 210023, People's Republic of China.

^*^ Corresponding author: Chuanfeng Tang

^**^ Corresponding author: Feng Zhou

E-mail address: tangchuanfeng@njucm.edu.cn (C.T.); zfibcas@163.com (F.Z.)

This file include:

Supplementary Information

Figure S1 and Figure S2

Table S1 and Table S2


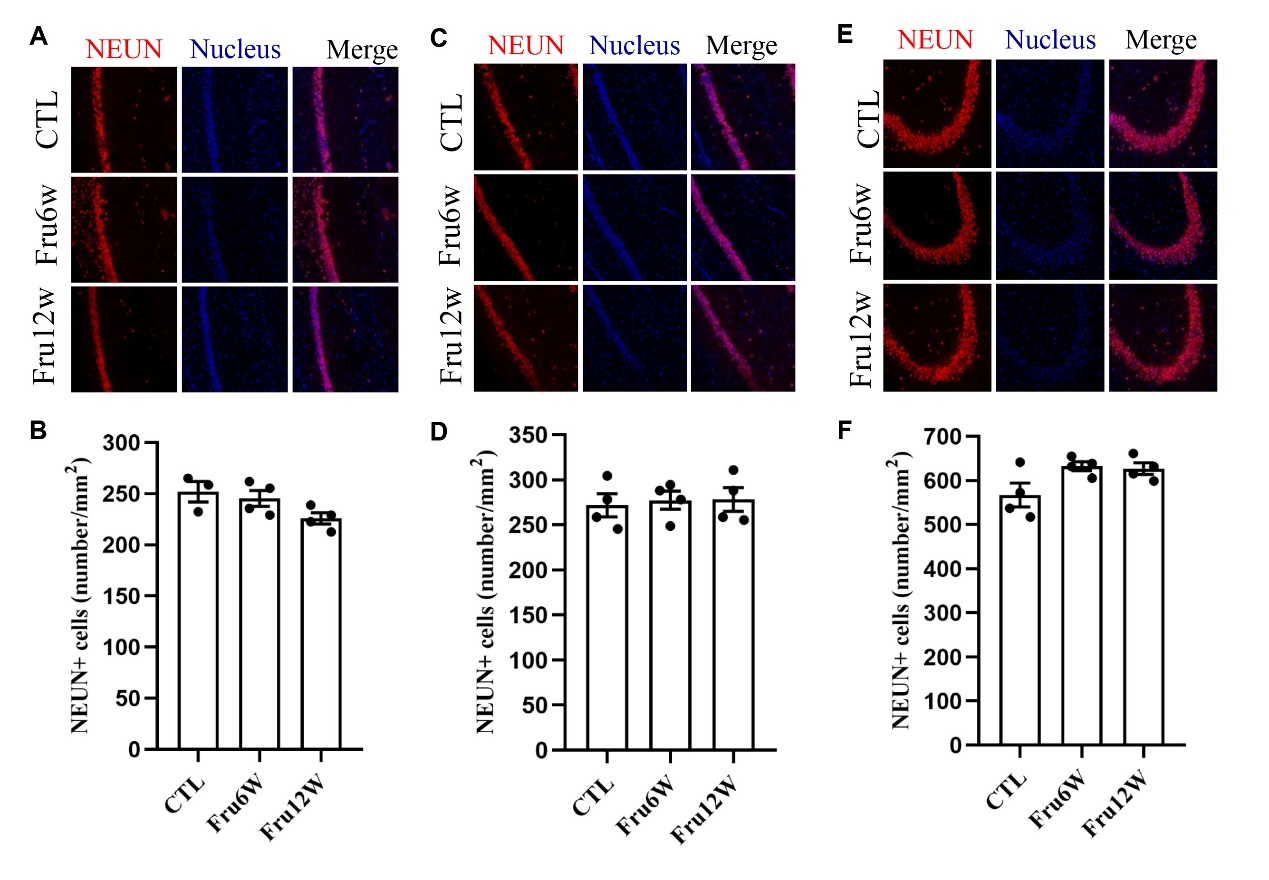
**Supplementary Information**

**Supplementary Figure 1.** **Immunofluorescent staining of mature neurons in the CA1, CA2, and CA3 regions of the mouse hippocampus.** (A) Representative images of NEUN markers in the CA1 region. (B) Statistical plot of the number of mature neurons in the CA1 region. (C) Representative images of NEUN markers in the CA2 region. (D) Statistical plot of the number of mature neurons in the CA2 region. (E) Representative confocal image of NEUN markers in the CA3 region. (F) Statistical plot of the number of mature neurons in the CA3 region. Data are expressed as Mean ± Sem. CTL: control group, Fru6W: high fructose diet 6 weeks group, Fru12W: high fructose diet 12 weeks group. CTL: control group, Fru6W: 6-week HFrD group, Fru12W: 12-week HFrD group.


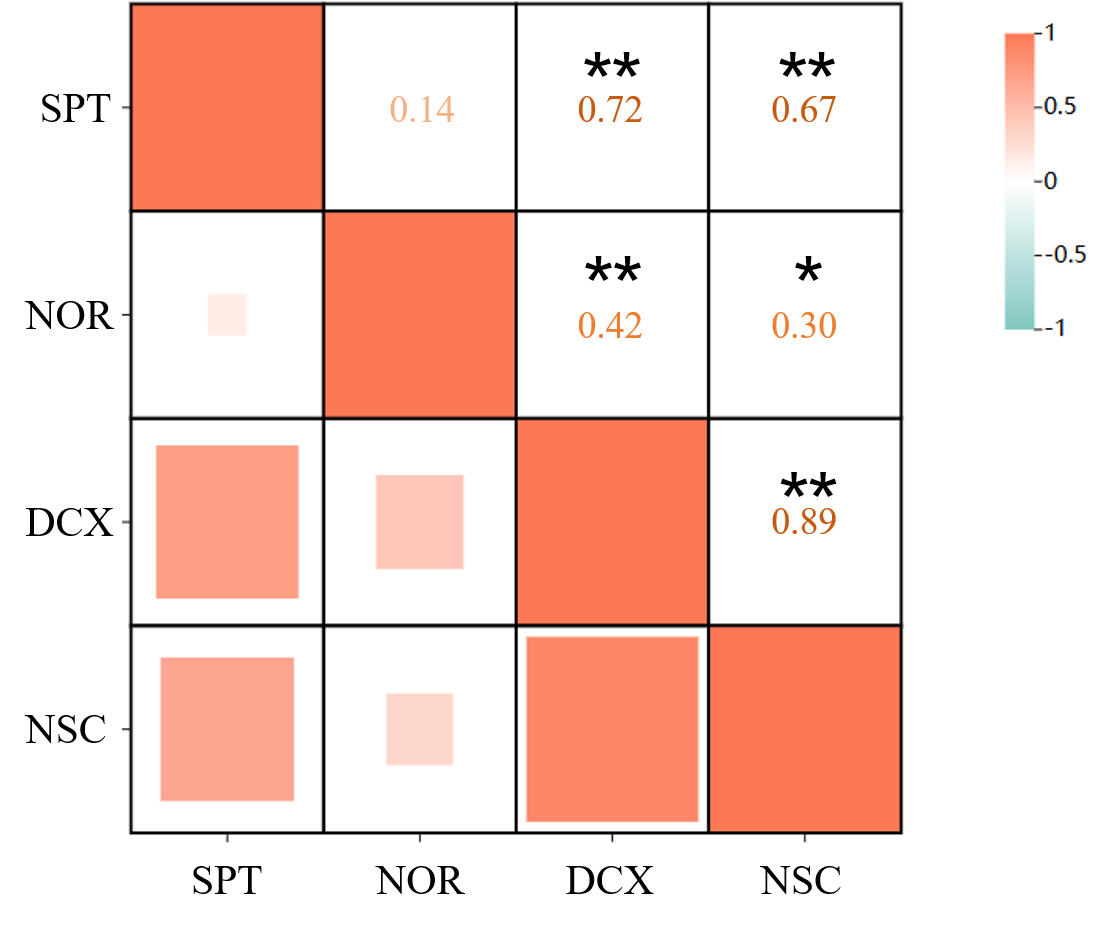


**Supplementary Figure 2. Correlation Heatmap Between Mouse Behavior and Neural Cell Count.** ** indicates a significant correlation at the 0.01 level (two-tailed), and * indicates a significant correlation at the 0.05 level (two-tailed). A “-” sign denotes a negative correlation, while the absence of “-” indicates a positive correlation. The strength of correlation is interpreted as follows:Very strong correlation: 1.0 > |R| ≥ 0.8; Strong correlation: 0.8 > |R| ≥ 0.6; Moderate correlation: 0.6 > |R| ≥ 0.4; Weak correlation: 0.4 > |R| ≥ 0.2; No correlation: |R| ≤ 0.2. Correlation analysis was performed using IBM SPSS Statistics version 27.0.1, and the correlation heatmap was generated using the online platform available at https://www.chiplot.online/.

**Table S1. Primer sequences of genes related to energy metabolism.**

| Gene ID | Forward Primer（5’-3’） | Reverse Primer（5’-3’） |
| --- | --- | --- |
| Slc25a5 | GCCGCTATATAAGTCGGCCA | AGCGGCATCTGTCATGTTGA |
| Atp5g1 | TTCTCCAGCTCTGATTCGCTC | CCGGGAAATGACACTGGTCT |
| Atp5g2 | CAGTGGAGTTGAAGCGACCA | TGTCGATGTCCCTTGAAATGG |
| Atp5g3 | CTGGTATTGGAACAGTCTTTGGC | GATCAAGAACGCAACCATCAAAC |
| Atp5h | ATGGCTGGGCGTAAACTTG | CTGGCGTGGAAGGTCTCATT |
| Atp5e | CAGGCTGGACTCAGCTACATC | GTTCGCTTTGAACTCGGTCTT |
| Atp5j2 | GCTGCCGAGCTGGATAATGA | TGCCAGGACCATGCTAATCC |
| Atp8a1 | CGGAGGACAGTGTCGGAGAT | TGGTTGATGAAGATGGTTCTCAC |
| Atp8a2 | CCGGCCCGCATAATTTACCT | TCTGCTCATACAGGAATCGAGG |
| Atp8b2 | CCAGGTGAATAACCGTCATTCTC | CTGCGGTCTCTATGTAACACAG |
| Atp9a | TCTAGAAGGGCTCTGCGTTC | TGGTATCCACAAGGACAGGC |
| Atp13a3 | GAGTCAGTCACAACAGATGCG | TGCACAAGATACACCTTCATCC |
| Atp6voc | ACTTATCGCTAACTCCCTGACT | ACACCAGCATCTCCGACGA |
| Atp6vod1 | TTGAGGGTGCAGGTAGCAATC | CGATGTTGCGACATTCCTGTTC |
| Atp2a1 | AAAGTCCCTGCAGACATCCG | GACAGGGTCTGTGTGCTTGA |
| Atp2b1 | TGAAGGAGCTGCGATCCTCTT | CTGTTCCTGCTCAATTCGACT |
| Atp2b4 | GGAAAGAACGTGATACCTCCAAA | GGCTGCGATCTCTAGGATGATG |
| Atp2b2 | GAGCGATGGTACAGTCTGCG | CTACATGGTCGGGACAGCTC |
| Atp5K | GTTCAGGTCTCTCCACTCATCA | CGGGGTTTTAGGTAACTGTAGC |
| SLC2A5 | CCAATATGGGTACAACGTAGCTG | GCGTCAAGGTGAAGGACTCAATA |
| SLC2A7 | CACGCACTTTGAGCGACAC | CCCACTTATTGACCATCAGGC |
| SLC2A8 | CCCTTCGTGACTGGCTTTG | TGGGTAGGCGATTTCCGAGAT |
| SLC2A9 | AAGCTCAGTGAAAAGAACTCCG | AGGTTGTACCCGTAGAGGAAG |

**Table S2. Primer sequences of genes related to proliferation, apoptosis, and ferroptosis.**

| Gene ID | Forward Primer（5’-3’） | Reverse Primer（5’-3’） |
| --- | --- | --- |
| AKT1 | TCAGGATGTGGATCAGCGAGAGTC | AGGCAGCGGATGATAAAGGTGTTG |
| AKT2 | GCCGCCTGCCATTCTACAACC | GCCTCTGCTTTGGGTCCTTCTTC |
| AKT3 | AAAGTATGACGACGACGGCATGG | CGCTTGCAGAGTAGGAGAACTGAG |
| CyclinA2 | GCCTTCACCATTCATGTGGAT | TTGCTGCGGGTAAAGAGACAG |
| CyclinB1 | ACTCCCTGCTTCCTGTTAT | AAAATGAGAAGTCACAACC |
| CyclinD1 | GCGTACCCTGACACCAATCTC | CTCCTCTTCGCACTTCTGCTC |
| CyclinD3 | CCTCCTACTTCCAGTGCGTG | GGCAGACGGTACCTAGAAGC |
| CyclinE1 | AAGCGAGGATAGCAGTCAGC | TCTGGGTGGTCTGATTTTCC |
| CyclinG2 | TCATTTCGGAGAGGCTAGCTG | ATATCTAATAAGTGTGCTTCTAG |
| CyclinH | CCTCGGATAATAATGCTTAC | TCATAGCCTTTCCTCTTC |
| CDK1 | CGTTTGACATCTGGAGTATAGG | GCCACACTTCGTTGTTAGGA |
| CDK2 | CTGGTCTGTTCATCGTGGTTCA | CCTGGCTCATTGGTGGTACATT |
| CDK4 | ATGGCTGCCACTCGATATGAA | TCCTCCATTAGGAACTCTCACAC |
| CDK6 | GGCGTACCCACAGAAACCATA | AGGTAAGGGCCATCTGAAAACT |
| BAX | CGTGAGCGGCTGCTTGTCTG | ATGGTGAGCGAGGCGGTGAG |
| BCL-XL | AACAATGCAGCAGCCGAGAGC | CAGAACCACACCAGCCACAGTC |
| CASP3 | AGTGGGACTGATGAGGAGATGGC | ATGCTGCAAAGGGACTGGATGAAC |
| CASP9 | GTGAAGAACGACCTGACTGCCAAG | GAGAGAGGATGACCACCACAAAGC |
| P53 | ACCGCCGACCTATCCTTACCATC | GGCACAAACACGAACCTCAAAGC |
| PTEN | GGAAAGGGACGGACTGGTGTAATG | CGCCTCTGACTGGGAATTGTGAC |
| SMAC | AACCACCTACGCGCTGATTGAAG | TACCTGCCACACCTCATCTTCCTC |
| FTH1 | CAGCGAGGTGGCCGAATCTTC | AGCCAGTTTGTGCAGTTCCAGTAG |
| GPX4 | ATAAGAACGGCTGCGTGGTGAAG | TAGAGATAGCACGGCAGGTCCTTC |
| MAVS | TCTCTTGTCCATCTCAGTCCA | TTCCCGATGTGCCTGTAGGA |
| NCOA4 | ACCAGCCTAGAGGTGTGGAGATTG | GTCCTGATGGTTCTGGGCAAGC |
| NLRP6 | GACGAGAGGAAGGCAGAG | TGGTGATGAAGAGCAGGT |
| GAD1 | CTGGCGTCATTGTGGAACCTCT | TCTCACTCAGCTCCACAGAGGT |
| GAD2 | CCTTGCAGTGTTCAGCTCTCCT | GCCTTGTCTCCTGTGTCATAGG |
| RIG-1 | AGAACAAACCGGGCAAC | CATCAGCGACCGAGGTA |
| SLC3A2 | GGTGGTGCTCAACTTCCGAGATTC | CGCTGGCTGGCAGGCTTATG |
| YAP-1 | GGACTCCGAATGCAGTGTCTTCTC | GCCGCTGTCTGTGCTCTCATC |
| GCLC | CTACCACGCAGTCAAGGACC | CCTCCATTCAGTAACAACTGGAC |
